# The assessment of musculoskeletal disorders, quality of life, and comorbidities in older people in Bangladesh

**DOI:** 10.3389/fpubh.2023.1269444

**Published:** 2023-12-15

**Authors:** Sharmila Jahan, Rabiul Islam, Tania Rahman, Md. Feroz Kabir, Md. Waliul Islam, Kabir Hossain, K. M. Amran Hossain, Md. Zahid Hossain, Ehsanur Rahman, Sonjit Kumar Chakrovorty, Altaf Hossain Sarker, Golam Moula, Atqiya Antara, Shahid Afridi

**Affiliations:** ^1^Institute of Social Welfare and Research, University of Dhaka, Dhaka, Bangladesh; ^2^Department of Physiotherapy and Rehabilitation, Jashore University of Science and Technology (JUST), Jashore, Bangladesh; ^3^Department of Physiotherapy, Centre for the Rehabilitation of the Paralysed (CRP), Savar, Bangladesh; ^4^Department of Microbiology, Jashore University of Science and Technology (JUST), Jashore, Bangladesh; ^5^Department of Physiotherapy, Dhaka College of Physiotherapy, Dhaka, Bangladesh

**Keywords:** Rehab assessment, muskuloskeletal disorders, older people, quality of life, aging

## Abstract

Musculoskeletal disorders are debilitating conditions that significantly impact the state of health, especially in older people. The study, which employed a cross-sectional design and practical sampling, included 206 participants among them 124 (62.2%) were men and 82 (39.8%) were women, from all over Bangladesh with musculoskeletal issues of varying severity and impact. The mean age of the participants was 64.9 (SD 4.3). The study was carried out between January and June of 2022. The majority of participants experienced musculoskeletal pain. Back pain was the most commonly complained of symptom among the participants (74.9%). It was also common to have limited mobility as a result of arthritic change, which eventually affected daily activities like taking care of oneself. To improve the health of the older adult population, more studies must be conducted to identify the many factors that contribute to musculoskeletal issues. The development of effective prevention and rehabilitation programs must then be based on this knowledge.

## Background

Decreased adult mortality and fertility rates are responsible for the global aging population (1). The nineteenth-century demographic transition in affluent countries is currently reshaping society in developing and low-income countries (2). Bangladesh has entered the third phase of demographic transition from a high mortality-high fertility regime to a low mortality-low fertility one (3). The “demographic dividend,” where developing countries’ working-age population outnumbers their dependent age population, is an opportunity that arises from this period of population transition. The most problematic diseases have shifted from communicable to non-communicable, especially chronic musculoskeletal ailments, as the population ages. Musculoskeletal problems were the most common chronic non-communicable disease in Brazil (4). Chronic musculoskeletal problems affect quality of life, independence, and social involvement because pain is the main complaint (5). The costs of musculoskeletal discomfort are second only to those of cardiovascular illness (6). Low back pain, common in middle-aged and older people, affects work impairment, absenteeism, and costs (7). “Nonspecific pain,” even when confined to a specific place (e.g., lower back), is common (8). Healthcare providers struggle with diagnosis and treatment complexity. To create effective health policies that promote health during advanced age and prevent disabilities, one must understand the issue at hand. This study examines musculoskeletal diseases in older Bangladeshis from a physiological standpoint. Musculoskeletal disorders and disabilities affect older individuals worldwide, justifying our investigation. Musculoskeletal problems are associated with mobility loss in older adults worldwide. Healthcare systems should prioritize reversing physical impairment in seniors. It is easier to handle common musculoskeletal problems in Bangladesh’s older individuals by understanding their prevalence and patterns. In Bangladesh, where aged care is scarce, family members may view older people as invalid and unable to access vitalhelp, causing them to suffer until their death. Psychosocial factors can affect geriatric musculoskeletal ailments. Thus, understanding the physiological basis of prevalent musculoskeletal problems in older individuals is essential for optimizing management, lowering impairment, boosting independence, and improving quality of life. Objectives The goal of this study is to assess the musculoskeletal disorders, quality of life, and comorbidities in older adults in Bangladesh.

## Methodology

With an emphasis on disability status and quality of life, as well as demographic, biological, psychological, and social factors, this study aimed to gain a physiological understanding of musculoskeletal problems in older persons (60 years) in Bangladesh. Based on the assumption that 24% of Bangladeshi adults have MSK conditions at a precision level of 5% (9), 280 participants were needed for each reporting domain. Taking into account an 85% response rate, a design effect of 1.5, and four reporting domains (male–female and rural–urban). A total of 206 people from all around Bangladesh with musculoskeletal problems of various severity and impact participated in the study, which used a cross-sectional design and convenient sampling. The research was conducted between the months of January and June of 2022.

The following criteria were used for the diagnosis of the MSK conditions: 1. Rheumatoid arthritis: 2010 American College of Rheumatology (ACR)/European League Against Rheumatism (EULAR) Classification Criteria (10); 2. Spondyloarthritis (axial and peripheral): Ankylosing Spondylitis Assessment Study (ASAS) criteria (11); 3. Ankysosing spondylitis: Modified New York Criteria 1984 (12); 4. Psoriatic arthritis: Classification Criteria for Psoriatic Arthritis (CASPAR) criteria (13); 5. Knee osteoarthritis: ACR clinical classification criteria for knee osteoarthritis (OA) (14); 6. Systemic Lupus Erythematosus: ACR Revised Criteria for the Classification of Systemic Lupus Erythematosus 1997 Systemic Lupus Erythematosus (15); 7. Soft tissue rheumatism: Commonly included subacromial bursitis, epicondylitis, trochanteric bursitis, anserine bursitis, and fibromyalgia [23]; Exclusion criteria was endoprothesis, metabolic syndrome (diabetes), acute traumas, mental problems. Data was collected through interviews that covered topics such as participants’ demographics, the participants’ experiences with and viewpoints on musculoskeletal illnesses, the participants’ capacities to do daily tasks, and their overall quality of life. Information was gathered via in-person interviews and careful observation. Various statistical methods, including frequency and percentage distributions, as well as qualitative and quantitative analysis in tabular form, were applied to the data collection. Quality of life in older individuals was evaluated using the WHO Quality of Life Scale (WHOQOL-BREF). The IRB, the Bangladesh Physiotherapy Association (BPA), and participant informed consent from participants all played roles in ensuring the study complied with ethical standards. All participants were free to leave the study at any time without penalty. People over the age of 60 with musculoskeletal diseases who did not match the inclusion criteria or who could not provide informed consent were not considered for participation. Hospitals and rehabilitation clinics all around Bangladesh helped fund the study, which was run by the Institute of Social Welfare and Research at the University of Dhaka. In conclusion, this study contributes significantly to our understanding of musculoskeletal problems from a physiological standpoint among the older adult population in Bangladesh.

### Statistical analysis

After being input into an Excel spreadsheet, the data were moved to EpiInfo (version 7) for analysis. In order to verify the denominators, missing values were found, and consistency was examined. Prior to analysis, all quantitative variables were categorized, including age, years of education, body mass index (BMI), and ADL score. Including those for MSK illnesses, impairments, and associated employment loss, 95% confidence intervals (CI) were computed. The findings were displayed for four reporting domains: sex groups, and residence locations in both urban and rural areas. For 11 potential variables (age, sex, education, wealth quartiles, urban location, smoking, rigorous physical activity, occupation, overweight, history of physical trauma, and diabetes), univariate logistic regression analysis was performed in order to get odds ratios (ORs).

## Results

**Figure 1:**
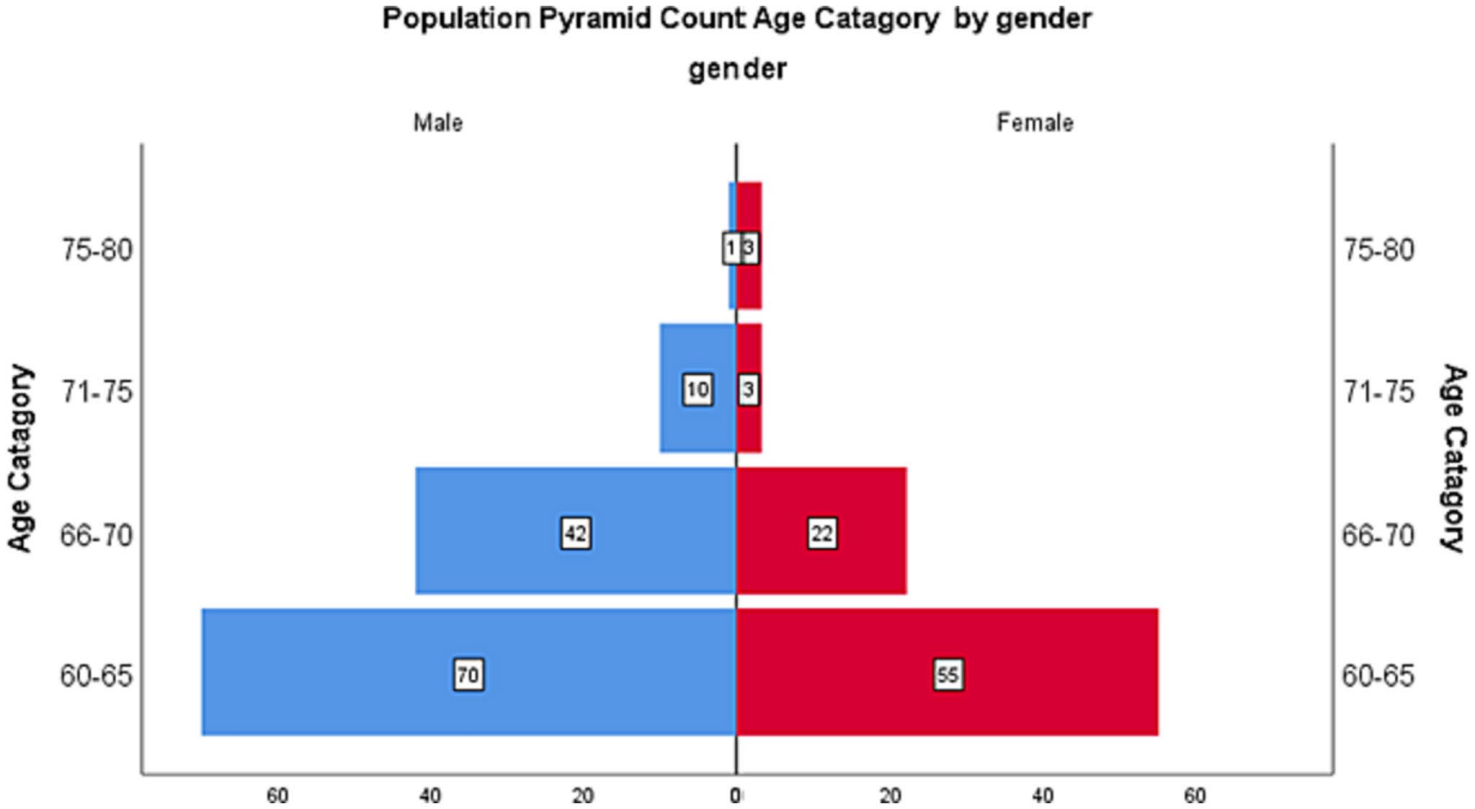
Population pyramid by gender and age.

Table 1 provides the socio-demographic characteristics of study participants. The table shows participants’ age, gender, dwelling area, marital status, Covid-19 status, and monthly income. Participants and variable percentages are shown. Most participants (60.7%) were 60–65 years old and married (88.3%) Figure 1.

**Table 1:** Socio-demographic characteristics of the participants.

	Total		Male		Female		*value of p*
	*n*	%	*n*	%	*n*	%	
*Age (years)*							
60–65	125	60.7	71	57.3	54	65.9	0.456
66–70	64	31.1	42	33.9	22	26.8	
71–75	13	6.3	10	8.1	3	3.7	
76–80	4	1.9	1	0.8	3	3.7	
*Living area*							
Semi-rural	76	36.9	15	12.1	10	12.2	0.789
Semi-urban	25	12.1	18	14.5	22	26.8	
Rural	40	19.4	44	35.5	32	39	
Urban	65	31.6	47	37.9	18	22	
*Marital status*							
Single	7	3.4	4	3.2	3	3.7	0.567
Married	182	88.3	115	92.7	67	81.7	
Married single	4	1.9	1	0.8	3	3.7	
Widow	7	3.4	0	0	7	6.1	0.345
Divorce	3	1.5	0	0	3	3.7	
Separated	2	1	2	1.6	0	0	
*Covid-status*							
Yes	37	18	20	16.1	17	20.7	0.455
No	169	82	104	83.9	65	79.3	
*Monthly income*							
<10,000	84	40.8	44	35.5	40	48.8	
10,000–24,999	47	22.8	29	23.4	18	22	0.453
25,000–49,999	26	12.6	18	14.5	8	9.8	
50,000–999,999	19	9.2	11	8.9	8	9.8	
≥100,000	30	14.6	22	17.7	8	9.8	

The gender distribution of participants in the table can be used to determine if men and women differ significantly. Table 2 shows participant musculoskeletal problems. The table lists back, neck, knee, heel, hip, gross muscle, elbow, wrist, and fracture statistics. The number of participants who reported each ailment and gender breakdown are shown. The table also includes Chi-square values and value of ps to investigate gender and musculoskeletal problems.

**Table 2:** Musculoskeletal disorder among participants.

Condition	Total		Male		Female		*X*^2^	*p*-value
	*n*	%	*n*	%	*n*	%		
Back pain	159	77.6%	92	74.8%	67	81.7%	1.582	0.208
Neck pain	84	41.0%	53	43.1%	31	37.8%	0.498	0.48
knee pain	173	84.4%	100	81.3%	73	89.0%	2.576	0.108
Heel pain	23	11.2%	14	11.4%	9	11.0%	0.005	0.944
Hip pain	13	6.3%	8	6.5%	5	6.1%	0.01	0.919
Gross muscle pain	26	12.7%	19	15.4%	7	8.5%	2.061	0.151
Elbow pain	39	19.0%	26	21.1%	13	15.9%	0.841	0.359
Wrist pain	52	25.4%	27	22.0%	25	30.5%	1.986	0.159
Fracture	4	2.0%	4	3.3%	0	0.0%		
Shoulder pain	30	14.6%	17	13.8%	13	15.9%	0.182	0.669

**Figure 2:**
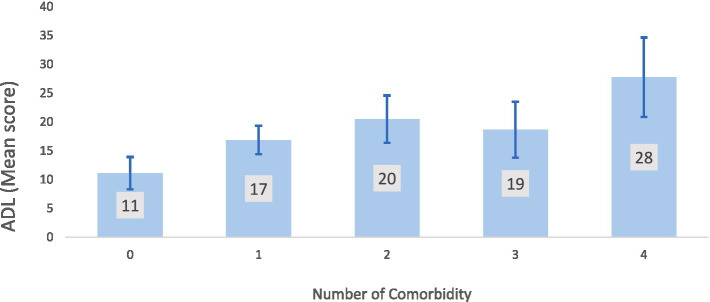
Mean ADL score of the participants according to number of comorbidity.

Figure 2 indicates that an older adult with four comorbidities has the highest score for activity limitation, whereas a person with no comorbidity has a mean activity limitation score of only 11. The chart illustrates that people with four comorbidities had the highest activity limitation score, while those without any had a mean score of 11. The more comorbidities, the more activity constraints. Table 3 shows the association between quality of life and activities of daily living. The table shows Pearson’s correlation coefficients between WHO-QoL BREF scores for physical, psychological, and social connection, and environmental domains and basic, instrumental, and typical social roles. Correlation coefficients measure two variables’ strength and direction. The table indicates a substantial positive correlation (0.95) between basic and instrumental activities of daily life, demonstrating that a person who can perform basic activities can also perform instrumental ones. The table also demonstrates negative associations between ADL measurements and physical and psychological domains of QoL scores, suggesting that increased activity limitation is related to lower QoL.

**Table 3:** Correlation between activity of daily living scale and quality of life (Preason’s Correlation).

	Basic activities of daily living (ADL)	Instrumental activities of daily living (ADL)	Participation in usual social roles	WHO-QoL BREF (Physical)	WHO-QoL BREF (Psychological)	WHO-QoL BREF (Social relationship)	WHO-QoL BREF (Environmental)
Basic Activities of Daily Living (ADL)	1						
Instrumental Activities of Daily Living (ADL)	0.9500	1					
Participation in Usual Social Roles	0.9289	0.9751	1				
WHO-QoL BREF (Physical)	−0.4073	−0.4550	−0.4472	1			
WHO-QoL BREF (Psychological)	−0.2711	−0.2473	−0.2368	0.3887	1		
WHO-QoL BREF (Social relationship)	−0.3455	−0.3564	−0.3526	0.7788	0.5677	1	
WHO-QoL BREF (Environmental)	−0.4156	−0.4318	−0.4274	0.8881	0.6533	0.8476	1

## Discussion

Older adults suffer from musculoskeletal problems that cause pain and disability. Neck, hip, knee, and lower back pain were reported by 98% of older persons (16). One-third had knee joint restrictions, 84% of respondents had weak muscles, and 78% struggled with daily tasks (17). Musculoskeletal and neurological problems that decrease muscle function cause geriatric falls. Physical disability, musculoskeletal issues, and inactivity increased falls. Western European and Japanese populations are aging, raising healthcare problems. Study heterogeneity affects older musculoskeletal disorder prevalence. Women report musculoskeletal pain more than men. Prevalence estimates tend to drop in the oldest age group (80+) (16). Healthcare practitioners must learn more about older people’s health and disabilities due to their growing numbers. Most older adults complained of joint pain, especially knee pain. Low back, shoulder, and neck pain were also common. Over 70% of seniors report some kind of discomfort (18). Southern India’s third most common complaint was back and neck pain. Older individuals’ musculoskeletal issues include joint degeneration and a restricted range of motion (19). Hip, lower back, neck, shoulder, and ankle joints were the most restricted. Knee pain was the most common complaint, followed by lower back, neck, shoulder, and ankle discomfort. Joint mobility and pain were linked (20). Older folks, especially women, were disabled because of arthritis. Knee pain was the most common musculoskeletal symptom in older adult women in this study, at 84%, while 25% of subjects reported wrist pain. Shoulder pain was reported by 14.6% of participants, but neck pain by 50% (21). Optimal healthcare is needed to keep older adults functional. Musculoskeletal problems in the aging population must be better understood due to their rising prevalence. The study was not free from limitation as the data were collected using in-person interviews and not standardized and validated questionnaires of MSDs, so the results of this study are difficult to compare with previous international studies. Additionaly, the physical activity of the participants was not assessed, and therefore it is difficult to analyze the results of quality of life.

## Conclusion

Nearly all participants had musculoskeletal pain. It was also typical to have restricted mobility due to arthritic change, which eventually has an impact on daily tasks like self-care. More research must be done to uncover the numerous elements that link to musculoskeletal problems in order to enhance the health of the older female population. This research must then be used to create successful programs for both prevention and rehabilitation.

### Ethical consideration

The proposal was approved by the Institutional Review Board (IRB) of the Institute of Physiotherapy, Rehabilitation, and Research of Bangladesh Physiotherapy Association (BPA-IPRR/IRB/19/01/2021/65). Written consent was taken from each participant before collecting the data. Informed consent was taken verbally during the phone call and written during the household survey. To ensure confidentiality, ethics, and privacy, the Declaration of Helsinki principles (22) were maintained throughout the research. One of our research team members obtained household screening approval from the Directorate General of Health Services of the Government of the People’s Republic of Bangladesh.

## Data availability statement

The raw data supporting the conclusions of this article will be made available by the authors, without undue reservation.

## Ethics statement

The studies involving humans were approved by Institute of Physiotherapy, Rehabilitation, and Research of Bangladesh Physiotherapy Association. The studies were conducted in accordance with the local legislation and institutional requirements. Written informed consent for participation was not required from the participants or the participants’ legal guardians/next of kin in accordance with the national legislation and institutional requirements.

## Author contributions

SJ: Conceptualization, Investigation, Project administration, Writing – original draft, Writing – review & editing. RI: Conceptualization, Investigation, Supervision, Writing – review & editing. TR: Conceptualization, Supervision, Writing – review & editing. FK: Conceptualization, Formal analysis, Funding acquisition, Methodology, Resources, Writing – original draft. WI: Data curation, Formal anlaysis, Funding acquisition, Investigation, Methodology, Project administration, Resources, Software, Visualization, Writing – original draft, Writing – review & editing. KaH: Investigation, Methodology, Visualization, Writing – original draft. KMH: Data curation, Methodology, Project administration, Writing – original draft, Writing – review & editing. ZH: Project administration, Writing – original draft. ER: Conceptualization, Writing – original draft. SC: Investigation, Writing – original draft. AS: Investigation, Writing – original draft. GM: Resources, Writing – original draft. AA: Resources, Writing – original draft. SA: Software, Writing – review & editing.
